# Physiological Effort in Submaximal Fitness Tests Predicts Weight Loss in Overweight and Obese Men with Prostate Cancer in a Weight Loss Trial

**DOI:** 10.23937/2378-3419/1410083

**Published:** 2017-10-16

**Authors:** Andrew D Frugé, John A Dasher, David Bryan, Soroush Rais-Bahrami, Wendy Demark-Wahnefried, Gary R Hunter

**Affiliations:** 1 Department of Nutrition Sciences, University of Alabama at Birmingham (UAB), USA; 2 UAB School of Medicine, Birmingham, Alabama, USA; 3 Department of Urology, UAB School of Medicine, Birmingham, Alabama, USA; 4 Department of Human Studies, UAB, Birmingham, Alabama, USA

**Keywords:** Prostatic neoplasms, Weight loss, Obesity, Aerobic fitness, Exercise

## Abstract

**Background:**

Obesity and weight gain after the diagnosis of prostate cancer are associated with an increased risk of prostate cancer recurrence and mortality; individualized plans to help prostate cancer survivors maintain or lose weight may be beneficial for recurrence risk reduction. Herein, we explore whether gains in cardiovascular fitness predict successful weight loss in men participating in a weight loss trial (NCT01886677).

**Methods:**

Forty men were randomized to receive twice-weekly in-person and telephone-based guidance on calorie-restricted diets and aerobic exercise to promote ~0.91 kg/week weight loss, or wait-list control. Thirty-two men completed submaximal VO_2_ Treadmill Tests (TT), anthropometric measures and two 24-hour dietary recalls at baseline and follow-up. For this secondary analysis, study arms were combined and associations between baseline and longitudinal changes in physiological effort (PE, measured by heart rate during TT), predicted VO_2max_, caloric intake and weight loss were analyzed.

**Results:**

Men lost 3.4 kg in 50 ± 23 days on the study. Multivariate linear regression indicated weight change was associated with change in PE at stage 2TT (Partial R = 0.635, p < 0.001), days on study (Partial R = −0.589, p = 0.002) and change in caloric intake (Partial R = 0.457, p = 0.019).

**Conclusions:**

Untrained men experiencing elevated heart rates during stage 2TT at baseline were able to achieve greater weight loss over the study period; this association was strengthened by a decrease in PE at the same level from baseline to follow-up concomitant with reduced caloric intake. Therefore, for these middle-aged and older men with lower aerobic fitness, exercise appears to be a key factor in achieving higher degrees of weight loss.

## Introduction

In 2016, roughly 180,890 men were diagnosed with prostate cancer, and 26,120 men died from the disease [1]. Obesity is a known risk factor for aggressive prostate cancer and is associated with higher risk of recurrence [2,3]. Because obesity has increased at a greater rate in cancer survivors than the general United States adult population in recent years, with 2014 estimates indicting that 31.7% of cancer survivors are obese [4], practitioners are encouraged to support weight loss efforts that are both effective and sustainable for their patients [3]. Not surprisingly, the American Cancer Society guidelines on nutrition and physical activity for cancer survivors’ first recommendation is to achieve and maintain a healthy weight by reducing high energy foods and increasing physical activity [5].

Lifestyle interventions in prostate cancer survivors have resulted in improved lifestyle behaviors [6], physical function, and quality of life [7]. Over the past two decades, guidelines for the treatment of overweight and obesity have endorsed the need for weight loss interventions to include guidance on diet, exercise and behavioral modification [8]. A recent review of weight loss interventions in prostate cancer survivors found that combined diet and exercise interventions were more effective than exercise alone for weight loss [9], though weight loss achieved through diet or exercise has shown similar benefits in chronic disease risk [10], inflammatory cytokines [11,12], and angiogenesis markers [13] in middle-age and older adults. It is known that caloric expenditure varies widely for similar activities in this study population; therefore, identifying predictive factors for better estimation of the energy costs of exercise is a priority set forth by the American College of Sports Medicine (ACSM) [14].

In previous studies, we have found that ease in locomotion during walking is positively related to total energy expenditure, activity related energy expenditure and non-exercise training activity related thermogenesis as measured with doubly labeled water after weight loss in overweight premenopausal women [15]. We have also found that ease of locomotion is inversely predictive of subsequent one-year weight gain [16]. Finally, we have shown that exercise training, either strength or aerobic training, induces improvements in ease of walking [17–19]. To our knowledge no one has studied the relationship between ease of locomotion, or its influence on weight change in men with prostate cancer. Herein, we pursue a secondary analysis of a randomized weight loss intervention in which there was substantial drop-in (voluntary weight loss) in the control group. We hypothesize that ease of locomotion, or physiological effort, will be associated with increased weight loss in men with prostate cancer participating in a presurgical weight loss trial.

## Methods

### Setting and participants

Men in this study had localized prostate cancer and participated in a presurgical weight loss trial between their time of diagnosis and radical prostatectomy [20]. Informed consent was obtained from all participants enrolled in the study, which was approved by the University of Alabama at Birmingham Institutional Review Board. Study participants had to have a Body Mass Index (BMI) > 25 kg/m^2^ and have no medical conditions affecting weight status or physical activity ability, have received no other treatment for their prostate cancer, and have surgery occurring at least 23 days after study enrollment.

### Intervention

Men completed all measures at their initial visit prior to randomization to the weight loss arm or a wait-listed control arm. The weight loss arm received guidance from a registered dietitian on a nutritionally adequate energy-restricted diet and daily aerobic exercise was prescribed and supervised by an exercise physiologist with the goal promoting 0.91 kg per week over the duration of the study [20].

### Measures

**Figure 1:**
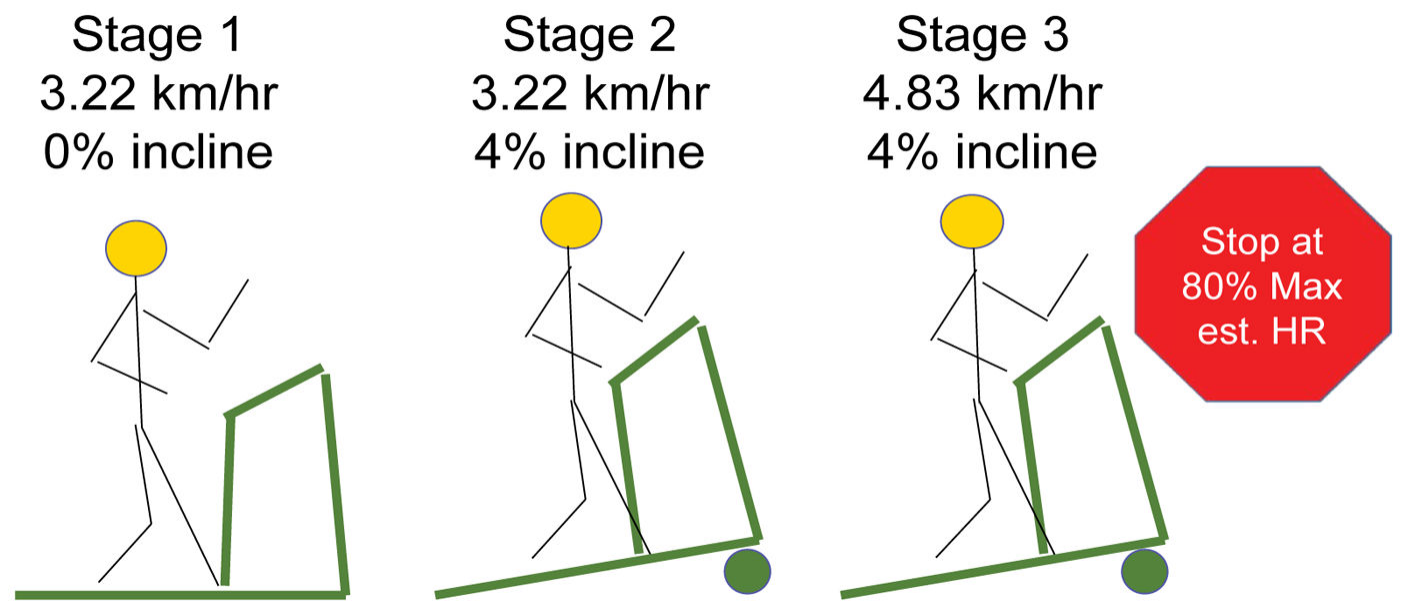
Baseline and follow-up VO_2submax_ treadmill tests were completed by men with prostate cancer participating in a presurgical weight loss trial. If 80% of estimated maximum heart rate was not reached in Stage 3, the following stages ensued: Stage 4) 6.44 km/hr, 4% incline; Stage 5) 6.44 km/hr, 8% incline.

Men completed dual energy x-ray absorptiometry (Prodigy, Lunar Radiation, Madison, WI), anthropometric measures and VO_2submax_ Treadmill Tests (TTs) during their initial and follow-up visits. The TT began with the participant seated for five minutes. Heart Rate (HR), oxygen consumption, ventilation, and the respiratory exchange ratio were monitored continuously from the seated position prior to and during TTs, which progressed in four minute stages as follows: Stage 1) 3.22 km/hr, 0% incline; Stage 2) 3.22 km/hr, 4% incline; Stage 3) 4.83 km/hr, 4% incline; Stage 4) 6.44 km/hr, 4% incline; Stage 5) 6.44 km/hr, 8% incline. Estimated maximum HR was calculated by subtracting the participant age in years from 220 (Figure 1). When the participant reached 80% of estimated maximum HR per ACSM criteria, the assessment was complete [21]. Time was noted and treadmill speed and incline were lowered slowly to assure participant safety. Physiological Effort (PE) was defined as HR at the end of each TT stage.

The predicted VO_2max_ was estimated using the following equations validated in a similar population with a correlation of 0.66 and an estimate of VO_2max_ within 0.5 mlO_2_/kg/min: Predicted VO_2max_ = 9.89 + 0.158 * est. max HR (220 - age) + 0.478 Stage 2 TT VO_2_ - 0.154 * Stage 2 TT HR.

Lean mass VO_2max_ = 9.89 + 0.158 * est. max HR (220 - age) + 0.478 Stage 2 TT VO_2_ - 0.154 * Stage 2 TT HR * Bodyweight/Fat-free mass.

Two-24 hour dietary recalls on non-consecutive days were obtained by a registered dietitian at both baseline and follow-up and entered into ASA24 (2011. Bethesda, MD: National Cancer Institute) [22,23], which derives caloric content of foods from the United States Department of Agriculture Food and Nutrient Database for Dietary Studies [24]. Average total calories at each time point were used for analyses.

### Statistical analysis

One-way analysis of variance was used to determine differences between fitness test variables between study arms. Due to substantial drop-in by the waitlist group, the entire sample was further analyzed as a whole. Differences in anthropometric and fitness variables from baseline to follow-up were compared using paired t-tests. Pearson bivariate correlations were used to explore relationships between weight change and fitness test variables. Multiple linear regression was used to examine the independent effects of change in HR at stage 2 of the fitness test, days on study, and change in caloric intake on weight change. All statistical tests were conducted using IBM SPSS Statistics, Version 22.0 (IBM Corp, Armonk, NY) and Bonferroni adjustment was used for multiple comparisons. Tests were considered statistically significant with a predetermined alpha of 0.05.

## Results

### Participants

Thirty-two men completed baseline and follow-up treadmill tests and were included in this analysis. Participants had a mean age of 60, ranging from 51 to 73 years. Twenty (62.5%) men were Caucasian and 12 (28.5%) were African American. Fourteen men were overweight (BMI 25 kg/m^2^ – 29.9 kg/m^2^) and 18 were obese (BMI ≥ 30 kg/m^2^) at baseline. At follow-up, only 11 men remained obese.

### Baseline and follow-up measures of adiposity and submaximal fitness tests

Data are shown in Table 1. Fifteen of the 32 men in this secondary analysis were randomized to the weight loss group; however, the between-arm difference in weight change did not achieve significance (p = 0.058). The combined sample of men included in this analysis lost an average of 3.4 kg over 50 ± 23 days on study.

Men had low baseline aerobic fitness as calculated by VO_2max_ (25.0 mlO_2_/kg/min) and lean mass VO_2max_ (42.0 mLO_2_/kg lean mass/min). Over the course of the study, participants increased VO_2max_ (0.8 ± 1.5 mlO_2_/kg/min, p = 0.007) with no change in lean mass VO_2max_ (p = 0.147). Measured HR and HR adjusted as a percentage of estimated maximum (220-age in years) slightly decreased from baseline to follow-up at stage 1 TT and stage 2 TT. No differences were observed between arms for changes from baseline to follow-up for PE or HR at any stage or predicted VO_2max_ (data not shown).

### Weight loss correlates

**Figure 2:**
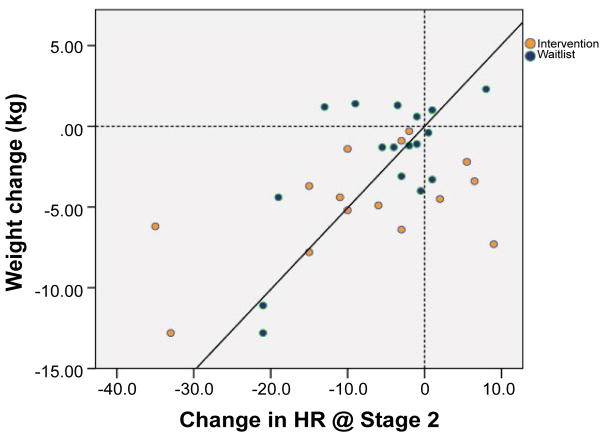
Weight loss correlates with decrease in heart rate in submaximal treadmill test Stage 2 TT (p = 0.0006) in men with prostate cancer participating in a presurgical weight loss trial (n = 32).

Pearson correlation coefficients for weight change, physiological effort and VO_2max_ are shown in Table 2. Weight loss was inversely associated with PE at stage 2 of baseline TT (p = 0.032) and positively correlated to baseline VO_2max_ (p = 0.021), indicating men who lost weight had a large HR response to physical exertion and low aerobic fitness at baseline. Adjusting VO_2max_ for lean body mass, weight change was not significantly correlated (p = 0.837). Changes in weight from baseline to follow-up were positively correlated to PE at stage 1 TT (p = 0.026) and stage 2 TT (p = 0.0006) (see Figure 2). However, change in estimated VO_2max_ was not associated with weight change (p = 0.184).

Correlates of weight change and PE adjusted as a percentage of maximum HR are shown in Table 3; correlation coefficients and p-values are similar to unadjusted PE variables in Table 2. The multiple regression model for weight change is presented in Table 4 indicating change in PE at stage 2TT (decrease in heart rate predicting increased weight loss), days on study (more days in study predicting increased weight loss), and change in caloric consumption (greater caloric reduction predicting greater weight loss) were independently related to weight change.

## Discussion

This is the first study exploring predictors of weight loss in men with prostate cancer. Despite small sample size, a reduction in HR during fitness testing predicted both a clinically and statistically significant weight loss, independent of age or baseline body mass; a finding that may help tailor future weight loss interventions in this population.

We hypothesized that physiological effort would be associated with weight loss; however these results suggest a much more dynamic relationship than predicted. The men exhibiting the highest levels of PE at baseline were more likely to lose weight over the course of the study. Physiological effort, or ease of locomotion, has previously been defined as a composite of fitness test HR, ventilation, and perceived exertion [25,26]. In this study, an increased HR at baseline alone preceded significant weight loss. This finding suggests there may be a potential disconnect between physiological and perceived effort in this population that may warrant further examination.

Participants in this study had baseline estimated VO_2max_ similar to a cohort of prostate cancer survivors in an exercise intervention that yielded increases in VO_2max_ over ten weeks in both low and high intensity exercise regimens [27]. This sample of prostate cancer survivors may not have attained similar increases in VO_2max_ due to the wide range (3-to-13 weeks) of time on the study. We adjusted our estimates of VO_2max_ to adjust for potential changes attributed to loss of fat mass but did not detect longitudinal changes using this metric. Also, submaximal fitness testing was used in this study due to the increased risk of adverse events associated with the age and BMI status of the study participants. Recent research has reported that maximal cardiopulmonary exercise testing in localized prostate cancer survivors may have low reliability [25], yielding significant changes in VO_2max_ in 1-to-2 weeks. Results from this study indicate VO_2max_ may also not be an ideal measure of fitness for this population.

Men who lost the most weight experienced the greatest reductions in PE walking on an incline, i.e. had the greatest increase in ease of locomotion. The conversion of HR during the submaximal walks to percent of estimated maximum HR gives a better estimate of ease of movement, and thus improved the associations with PE and weight loss. This finding was consistent with results from our previous studies in which we have shown that ease in locomotion is related to increased total energy expenditure and activity related energy expenditure [17–19,28], as well as subsequent one year weight change in healthy subjects [16]. It is not known from these data whether improvement in ease of locomotion results in more physical activity and thus more weight loss, or whether weight loss causes an increase in ease of locomotion, or both. Future studies that assess PE at several time points throughout weight loss coupled with qualitative data may detangle this relationship. A 3-month physical activity intervention in breast cancer survivors significantly decreased the rate-pressure product, which positively associated with fatigue [26]; a link that may support ease of locomotion preceding more physical activity and potentially weight loss. Similarly, prostate cancer survivors in a 12-week training program significantly reduced their resting HR in comparison to controls [29], but decreases in resting HR were not observed for weight losers in this study.

In addition to the decrease in stage 2 PE positively associating with weight loss, multiple regression analysis indicated a significant relationship with days spent on study and change in caloric intake. When baseline VO_2max_ was added in to the regression, all other p-values did not change significantly; therefore, VO_2max_ at baseline was not a confounding factor, but experimental intervention (dietary and exercise guidance) was important for promoting more rapid weight loss. The combined effect of decreased PE and reduction in caloric intake reinforce the need for both physical activity and diet modification for successful weight loss.

Men in this randomized controlled trial were motivated to lose weight, regardless of study arm allocation. Though only a small sample, this reinforces the concept of the teachable moment in cancer diagnosis and lifestyle change, as well as the potential impact of clinician encouragement to achieve a healthy weight [3,30]. The most profound finding in this secondary analysis is the association between physiological effort and the ability or propensity to initiate and continue physical activity achieving clinically meaningful weight loss in a matter of weeks. The most probable explanation for this relationship is that men with high baseline PE at early stages of the treadmill test were able to achieve greater caloric expenditure once they began exercising. This enabled attainment of a substantial caloric deficit which resulted in self-reinforcing, marked weight loss with minimal to moderate perceived effort.

### Limitations

While this is the first study reporting clinically significant associations with weight loss and HR at low level exercise in men with prostate cancer, there are limitations. First, VO_2max_ was estimated rather than directly tested. Additionally, there are generalizability limitations inherent with a small sample size. Translational potential of this exploratory study relies on replication in an appropriately powered intervention with the goal of increasing the probability of weight loss success. Future studies should also evaluate weight loss maintenance for men with prostate cancer that achieve short-term success associated with decreased PE.

## Conclusion

In conclusion, this study provides data to support an alternative means of predicting successful short-term weight loss in men with prostate cancer. These results suggest that increasing exercise efficiency is strongly associated with weight loss. Though the direction of causality was not determined in this study, both exercise and weight reduction for overweight and obese prostate cancer survivors have known benefits. While decreasing caloric intake is essential for long-term weight loss, aerobic exercise may accelerate and/or amplify success for many of these older men; oncologists and other health care providers should encourage both caloric restriction and aerobic physical activity among the overweight and obese prostate cancer patients for which they provide care.

## Figures and Tables

**Table 1:** Baseline and follow-up measures for men with prostate cancer participating in a presurgical weight loss trial (n = 32).

	Baseline Mean ± SD	Follow-up Mean ± SD	Change Mean ± SD
Body weight (kg)	96.4 ± 13.8	93.0 ± 13.3	−3.4 ± 3.9***
Body Mass Index (kg/m^2^)	31.1 ± 4.4	30.0 ± 4.2	−1.1 ± 1.3***
Percent body fat^a^	36.0 ± 5.5	34.6 ± 5.5	−1.4 ± 1.9***
Caloric Intake^b^	1587 ± 525	1298 ± 432	289 ± 667*
Stage 1 VO_2_	10.1 ± 1.9	9.6 ± 2.0	−0.4 ± 1.7
Stage 2 VO_2_	13.1 ± 2.1	12.5 ± 2.2	−0.5 ± 2.2
Stage 1 HR	96.1 ± 13.3	90.1 ± 13.3	−6.0 ± 9.9**
Stage 2 HR	106.8 ± 14.2	100.1 ± 13.3	−6.7 ± 10.6**
Stage 1% of max HR	0.601 ± 0.086	0.563 ± 0.087	−0.038 ± 0.062**
Stage 2% of max HR	0.667 ± 0.088	0.626 ± 0.086	−0.041 ± 0.066**
Predicted VO_2max_	25.0 ± 2.0	25.8 ± 2.1	0.8 ± 1.5**
Predicted Lean mass VO_2max_^a^	42.0 ± 1.4	41.7 ± 1.4	−0.3 ± 1.1

a n = 31 due to no DEXA data;

b Average of two-24 hour dietary recalls, n = 28; Stage 1: 3.22 km/hr, no incline; Stage 2: 3.22 km/hr, 4% incline;

* p < 0.01;

** p < 0.05;

*** p < 0.001, significant at p < 0.004.

**Table 2:** Correlations between weight change, physiological effort and predicted VO_2max_ in men with prostate cancer participating in a presurgical weight loss trial (n = 32).

	Change in weight (kg)	Baseline HR @ Stage 1	Baseline HR @ Stage 2	Baseline VO_2max_	Baseline lean mass VO_2max_	Change in HR @ Stage 1	Change in HR @ Stage 2	Change in predicted VO_2max_	Change in lean mass VO_2max_
Change in weight (kg)	1	−0.260	−0.380*	0.406*	−0.039	0.393*	0.576**	−0.241	−0.058
Baseline HR @ Stage 1		1	0.943***	−0.818***	0.375*	−0.370*	−0.282	−0.009	−0.063
Baseline HR @ Stage 2			1	−0.791***	0.503**	−0.396*	−0.453**	−0.033	−0.107
Baseline Predicted VO_2max_				1	0.136	0.356*	0.341	−0.166	−0.101
Baseline lean mass VO_2max_					1	−0.134	−0.251	−0.264	−0.294
Change in HR @ Stage 1						1	0.826***	−0.154	−0.078
Change in HR @ Stage 2							1	−0.175	−0.059
Change in predicted VO_2max_								1	0.975**

Stage 1: 3.22 km/hr, no incline; Stage 2: 3.22 km/hr, 4% incline;

a n = 31 due to no DEXA data;

* p < 0.01;

** p < 0.05;

*** p < 0.001.

**Table 3:** Correlations between weight change and physiological effort as a percent of estimated maximum heart rate in men with prostate cancer participating in a presurgical weight loss trial (n = 32).

	Change in weight (kg)	Baseline % of Max HR @ Stage 1	Baseline % of Max HR @ Stage 2	Change in % of Max HR @ Stage 1	Change in % of Max HR @ Stage 2
Change in weight (kg)	1	−0.270	−0.398*	0.394*	0.583***
Baseline % of Max HR @ Stage 1		1	0.947***	−0.351*	−0.236
Baseline % of Max HR @ Stage 2			1	−0.385*	−0.410*
Change in % of Max HR @ Stage 1				1	0.823***

Stage 1: 3.22 km/hr, no incline; Stage 2: 3.22 km/hr, 4% incline;

* p < 0.01;

** p < 0.05;

*** p < 0.001.

**Table 4:** Multiple regression model of weight change in men with prostate cancer participating in a presurgical weight loss trial (n = 28).

	R	B	Standardized beta	Partial r	P value
Weight change (kg), p < 0.001	0.792				
Intercept		2.60			
Change in HR @ Stage 2		0.19	0.509	0.635	0.0005
Days on study		−0.08	−0.453	−0.589	0.0015
Change in caloric intake		0.002	0.315	0.457	0.0189
